# Pathological and clinical characteristics of late-onset oligomeganephronia based on a histomorphometric study

**DOI:** 10.1186/s12882-023-03096-3

**Published:** 2023-03-15

**Authors:** Ya-Li Ren, Yang Li, Jie Gao, Xu-Jie Zhou, Li Yang, Su-Xia Wang

**Affiliations:** 1 Laboratory of Electron Microscopy, Pathological Center, Peking University First Hospital, No. 8, Xishiku Street, Beijing, 100034 People’s Republic of China; 2 Renal Division, Department of Medicine, Institute of Nephrology, Key Laboratory of Renal Disease, Key Laboratory of CKD Prevention and Treatment, Peking University First Hospital, Peking University, Ministry of Health of China, Ministry of Education of China, Beijing, People’s Republic of China; 3 Department of Clinical Laboratory, Tongji Shanxi Hospital, Shanxi Bethune Hospital, Shanxi Academy of Medical Sciences, Third Hospital of Shanxi Medical University, Taiyuan, People’s Republic of China

**Keywords:** Chronic kidney disease, Glomerulomegaly, Histomorphometry, Adult, Oligomeganephronia

## Abstract

**Background:**

Late-onset oligomeganephronia (OMN) is a rare chronic kidney disease and has no quantitative criteria for diagnosis yet. The current study aimed to explore its clinicopathological features by histomorphometric analysis.

**Methods:**

We retrospectively re-reviewed all patients with enlarged and sparse glomeruli by light microscopy at Peking University First Hospital from 2012 to 2021, excluding those with any factor known to contribute to similar changes. Age- and sex-matched patients with thin basement membrane nephropathy were selected as control to establish the cut-off values for glomerulomegaly and rarity. Late-onset OMN cases were then confirmed and the clinicopathological characteristics were summarized.

**Results:**

Mean diameter and density of cortical glomeruli in control was 156.53 ± 27.50 μm and 4.07 ± 0.63 /mm^2^, giving a lower limit of 211.53 μm for glomerulomegaly and an upper of 2.81 /mm^2^ for rarity. Seven adults of three females and four males were finally diagnosed as late-onset OMN with a mean age of 26.57 years. They showed mild to moderate proteinuria and/or renal dysfunction at biopsy with the mean proteinuria, serum creatinine (Scr) level, and estimated glomerular filtration rate of 0.50 g/d (0.10–0.95 g/d), 140.9 µmol/L (95.1–227.1 µmol/L), and 58.7 mL/min/1.73m^2^ (21.3–98.0 mL/min/1.73m^2^), respectively. Four patients (57.1%) had normal Scr at diagnosis. Six patients with available data showed renal tubular injury with increased urinary microalbumin in all, elevated N-acetyl-β-glucosaminidase in two, and elevated α1 microglobulin in five. Kidney size was normal or slightly reduced. The mean density and glomerular diameter of the seven cases was 0.86 mm^2^ (0.55–1.41 /mm^2^) and 229.73 μm (211.88–260.66 μm). Segmental glomerular sclerosis was observed in six (85.7%) with four (66.7%) of perihilar type. Proximal tubule dilation was observed in all, focal to diffuse, lining with enlarged epithelial cells. The mean foot process width was 634.02 nm, wider than 472.54 nm of the control (*P* = 0.0002).

**Conclusion:**

Late-onset OMN should be considered a special entity with relatively slow clinical progress characterized by hypertrophy of the sparsely distributed nephron.

## Introduction

Oligomeganephronia (OMN), which is also called oligomeganephronic renal hypoplasia, is conditionally defined as a rare type of renal hypoplasia characterized by small kidneys with reduced number of renal lobes mainly occurs in infants or children [1]. Histologically, it shows a strikingly reduced number of nephrons which are markedly enlarged [1–3]. The diagnosis can only be confirmed by renal biopsy. So far, only a few reports can be obtained and most of them consist of case reports or small case series. In the only large series by Broyer et al. [4], a rigorous diagnostic criteria were suggested as follows. (i) Reduction of kidney size with the sum of both kidney lengths ≤ 80% of 1 kidney of the same-sized child. (ii) Reduction in glomerular filtration rate to 30% of normal. (iii) Absence of urinary tract malformations and no evidence of significant vesicoureteral reflux (small amounts of reflux were accepted provided no scars were identified). Since such a strict standard is difficult to meet in practice, many reports have employed far more loose criteria. In contrast to the above traditional early-onset OMN, similar pathological changes can occur in adults which is called late-onset OMN. As far as we know, there are only seven adult patients with OMN in English-published literature [2, 5–8], presenting different features from the traditional OMN patients in children. Moreover, the pathological diagnosis of late-onset OMN is still controversial due to the lack of definite quantitative criteria. In this study, by analyzing the data from the control patients of thin basement membrane nephropathy, we try to establish quantitative criteria for glomerular hypertrophy and rarity based on histomorphometric study. Using this standard, cases suspicious of late-onset OMN during the past ten years in our center were reassessed and seven of them were eventually diagnosed. Subsequently, the clinical and pathological manifestations of these patients were presented and the difference between early-onset and late-onset OMN were compared with literature review.

## Materials and methods

### Patients

Renal biopsy samples in Peking University First Hospital from 2012 to 2021 were re-reviewed. Those samples that presented with hypertrophic and scattered glomeruli, mild interstitial fibrosis and tubular atrophy (IFTA) of less than 25%, and with no obvious ischemic glomeruli were collected. All patients showed normal bilateral renal pelvis, calyces, ureters, and bladder, with no evidence of postrenal obstruction. Rule out criteria included the conditions that may lead to glomerulomegaly, such as smoking, obesity, diabetes, family history of end-stage renal disease (ESRD), or elevated serum uric acid level. In addition, any other type of histologically confirmed glomerular disease was also excluded. Age- and sex-matched control group with thin basement membrane nephropathy (TBMN) was obtained from percutaneous renal biopsies. Rule in criteria of the control group included: (i) The main clinical manifestation was asymptomatic hematuria; (ii) Proteinuria was absent or trace (< 0.15 g/24 h); (iii) For all patients, there were no abnormal renal function and no conditions listed above that may lead to glomerulomegaly; (iv) Except for thin glomerular basement membrane (GBM), the specimen had no evidence of other glomerular diseases by immunofluorescence (IF), light microscopy (LM), or electron microscopy (EM); (v) The proportion of globally sclerotic glomeruli was within the allowed range, less than the percentage calculated by subtracting 10 from half the patient’s age, namely < (age/2–10)%.

Informed consent was obtained from each patient for blood sampling and renal biopsy. The research was in compliance with the Declaration of Helsinki and was approved by the local ethics committee of Peking University First Hospital [IRB number 2017 − 1280].

### Clinical and laboratory parameters

Clinical and laboratory data at the time of renal biopsy including gender, age, body mass index (BMI), blood pressure, albumin, urinary protein per 24 h, hematuria, serum creatinine (Scr), creatinine clearance rate (Ccr), C3 level, urinary microalbumin, transferrin, N-acetyl-β-glucosaminidase (NAG), α1 microglobulin (α1M), immunoglobulin, the range of proteinuria and Scr during the course of the disease, and the past and family history were collected from the patients’ medical records. Glomerular filtration rate (GFR) was evaluated by estimated GFR (eGFR) using the Epidemiology Collaboration Equation [9]. The data provided by renal ultrasound examination including renal size, cortical thickness and other changes were obtained too. We also retrospectively collected the clinical data in follow-up after their biopsy.

### Histological evaluation

The renal biopsy specimens were processed using standard techniques for direct IF, LM, and EM. The samples were fixed in 4.5% buffered formaldehyde for LM and in 2.5% glutaraldehyde for EM, respectively. Consecutive serial sections were used for histological staining with hematoxylin and eosin, periodic acid-Schiff, silver methenamine, and Masson’s trichrome individually. EM was performed according to standard procedures. After embedded in Epon, ultrathin sections were mounted on copper grids and stained with uranyl acetate and lead citrate prior to being viewed by a transmission EM (JEM-1230 JEOL, Tokyo, Japan). Immunofluorescence staining was performed using antibodies against IgG, IgA, IgM, kappa light chain, lambda light chain, C3, and C1q. Two pathologists evaluated the biopsies separately and reach a consensus on the diagnosis.

### Image measurement

The images of the biopsy tissues were collected by LM (magnification of 100×). AnalySIS Image Processing software (Olympus Soft Imaging Solutions, Munster, Germany) was used to obtain the concerned indicators in each tissue. The following indicators including the density of glomeruli in the renal cortex, the diameter of glomeruli and proximal tubules (PT), the thickness of GBM and the height of epithelial cells lining PT were assessed.

#### Assessment of glomerular density and diameter

Glomerular density was obtained by dividing the number of all glomeruli by the area of renal cortex in each sample. The index of cortical area was got automatically from the software by drawing the outline of the obtained cortex. All glomeruli in the cortex including incomplete or obsolete ones were counted.

The mean glomerular diameter was used to represent the size of each glomerulus. Outline of the outer glomerular capillary loops of the tuft and the parameters would be calculated automatically by the software. All glomeruli were measured except those incomplete, those with sclerosis more than 25%, those with significant shrinkage of capillary loops even accompanied by periglomerular fibrosis, and those only with a few capillary loops and with no mesangium observed.

#### Assessment of diameter of PT and height of overlying epithelial cells

We randomly selected PT for measurement through the uniformly spaced test lines produced by the software over each photograph. Only those with non-atrophic intact hit by the test lines could be measured by delineating the outline profile along tubular basement membrane (TBM). In order to minimize the influence of different sections on the size of tubules, the shortest diameter which was given by the software was chosen as the index to represent the size of PT.

Intact profile of PT was unnecessary for the height of epithelial cells. The height was indicated by the shortest distance between the TBM and the corresponding luminal surface of the cells, a vertical line perpendicular to the TBM initiating from the intersection point of the test line and TBM to the luminal surface of the cell. It should not be measured when the luminal boundary of the cells was not clear due to shedding of the brush border, and confluence of adjacent or contralateral cells.

#### Evaluation of the thickness of GBM and the foot process width (FPW) of podocytes

By EM, the thickness of GBM was evaluated by the distance between the basal aspect of the endothelial membrane and the basal aspect of the podocyte membrane that touches the GBM. Measurements were made along uniform cross-sections of the glomerular capillary wall. FPW of podocytes was obtained to evaluate the degree of foot process effacement as described previously [10]. In brief, the arithmetic mean value of FPW was obtained by calculating the ratio of the length of GBM to the number of foot processes along it in at least ten randomly taken photos of each patient. Then it was corrected by the correction factor “π/4” for presumed random variation in the angle of section relative to the long axis of the podocyte [11]. Any foot process was estimated based on examination of all nonsclerotic glomerular capillaries in all fields.

### Statistical analysis

All analyses and calculations were performed with the SPSS 14.0 software (IBM, Armonk, NY, USA). The measurement data were expressed as mean ± standard deviation (SD) and/or range for continuous variables and as proportions for categorical variables. The significance between OMN group and the control were determined by Student’s t-test. Differences were considered significant when *P* < 0.05.

## Results

### Quantitative criteria for glomerular hypertrophy and rarity and accordingly confirmed OMN cases

There were eight cases in the control, three males and five females with an average age of 26 years (range 21–32 years) and an average BMI of 21.48 kg/m^2^ (19.71–23.12 kg/m^2^). A total of 96 glomeruli were measured and the mean diameter was 156.53 ± 27.50 μm (range 83.21–221.81 μm). Since the measurement data of diameter of glomerular capillary tuft in the control fit the normal distribution, mean ± 2SD was used for expression of the normal value range. Accordingly, the upper limit of diameter for normal glomerular of our controls was 211.53 μm. Similarly, the mean density of cortical glomeruli of the control cases was 4.07 ± 0.63/mm^2^ (range 3.21–4.92/mm^2^), giving a lower limit of 2.81/mm^2^. Therefore, the cut-off values for defining glomerular hypertrophy and rarity were an average glomerular diameter beyond 211.53 μm and a glomerular density below 2.81/mm^2^. The case that met with both criteria would be confirmed the diagnosis of OMN.

**Fig. 1:**
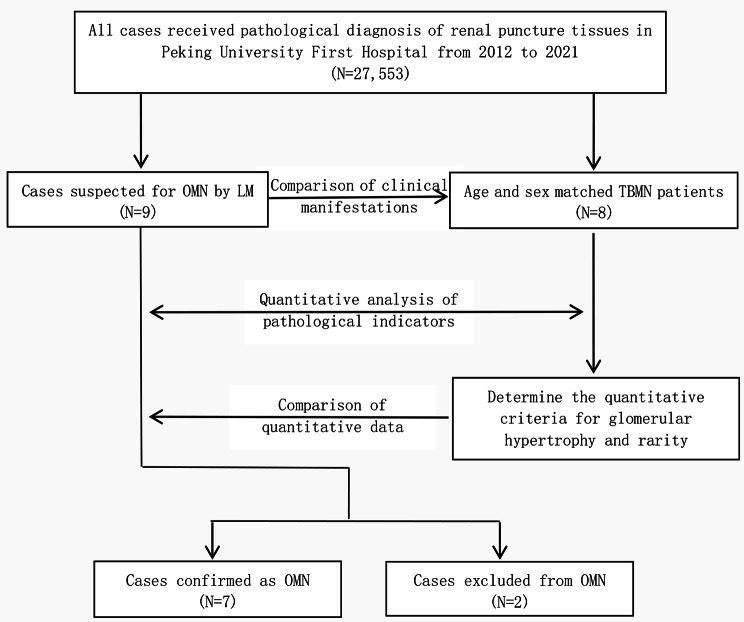
Data of OMN patients and TBMN patients were collected and analyzed. A total of 27,553 cases of kidney biopsy accessioned in our center in the past ten years. Nine cases were suspected for OMN by LM. According to the quantitative criteria of glomerular hypertrophy and rarity determined by the TBMN group, seven cases were finally confirmed as OMN while the other two were excluded. OMN: oligomeganephronia; LM: light microscopy; TBMN: thin basement membrane nephropathy

As shown in Fig. 1, there were 27,553 cases of kidney biopsy accessioned in our center in the past ten years. Nine cases were suspected for OMN by LM after excluding cases with other clinical factors that may lead to glomerular hypertrophy and those with other glomerular diseases, among which two cases did not meet the above quantitative criteria. Eventually, seven cases were confirmed as OMN. The initial and final dates of biopsies was August 2012 and August 2021 separately.

### Clinical presentation of OMN cases

The patients’ clinical characteristics were shown in Table 1. The cohort consisted of seven adults, three females and four males, with a mean age of 26.57 years (SD 5.83 years, range 19–38 years) and a mean BMI of 21.09 kg/m^2^ (SD 1.26 kg/m^2^, range 19.4–23.24 kg/m^2^). All seven cases presented with proteinuria and/or mildly impaired kidney function at the time of biopsy. The mean proteinuria, Scr level, and eGFR at biopsy was 0.50 g/d (range 0.1–0.95 g/d), 140.9 µmol/L (range 95.1–227.1 µmol/L), 58.7 mL/min/1.73m^2^ (range 21.3–98.0 mL/min/1.73m^2^), respectively. Four patients (57.1%) had normal Scr at diagnosis. Duration the illness of an averaged 24.6 months, proteinuria had ever reached 1.0 g/d in three patients with a maximum of 2.0 g/d, and the maximum of Scr was 240 µmol/L. All patients presented with no to trace hematuria. The minimum of Ccr was 29.1 mL/min/1.73m^2^. Albumin level was within normal limit in all patients. The data for tubule injury was obtained from six cases with increased urinary microalbumin in all patients ranged from 36 to 1300 mg/L, elevated urinary transferrin in five (range 2.27–83.7 mg/L), NAG in two (21 U/L and 44 U/L), and α1M in five (range 15.3–82.5 mg/L). C3 level was in normal range in all patients except one was slightly depressed. Renal ultrasound demonstrated that the size of the kidney was normal or slightly reduced with the right length ranged from 8.0 to 11.5 cm and the left length from 8.4 to 12.2 cm, all with enhanced echo of renal parenchyma. None of the patients was diagnosed with hypertension except one (Case 2) presented with transient elevated blood pressure, and none of them complained of history of intrauterine growth restriction, maternal diseases, maternal drug intake or premature birth.

**Table 1:** Clinical features of the seven late-onset OMN cases

Case No.	Case 1	Case 2	Case 3	Case 4	Case 5	Case 6	Case 7
Sex; Age (years)	M; 28	F; 19	M; 26	F; 27	M; 24	F; 38	M; 24
BMI (kg/m^2^)	20.2	21.0	23.24	19.4	20.53	22.03	21.26
Complaint							
PrU	Yes	Yes	Yes	No	Yes	Yes	Yes
Elevated Scr	No	Yes	No	Yes	Yes	Yes	No
BP (mmHg)	110/80	110/70	130/80	110/70	130/70	110/85	120/80
Albumin (g/L)	42.3	46.9	45.5	45.4	39.0	37.5	48.8
PrU (g/d)/UV (mL/d)	0.83/2200	0.10/1660	0.95/1800	0.21/1930	0.88/2750	0.22/1830	0.31/2400
HeU	NEG	NEG	NEG	NEG	Trace	NEG	NEG
Scr (µmol/L)	135.2	116.9	95.1	128.7	152.2	227.1	131.1
*PrU (g/d)	0.84–1.5	0.1–0.18	NA	NEG to 0.21	0.88–2.0	0.22–1.73	NA
*Scr (µmol/L)	130–147	107–184	NA	112–140	152–160	179–240	123–131
eGFR(mL/min/1.73m^2^)	63.0	60.0	98.0	49.1	54.4	21.3	65.1
Ccr (ml/min)	89.5	41.5	NA	58.3	74.4	29.1	63.8
Complement 3 (g/L)	NA	0.82	1.32	0.61	NA	0.48	0.80
MAU (mg/L)	1300	36	NA	48	291	64	239
TFU (mg/L)	83.7	< 2	NA	4.2	7.4	2.27	9.95
NAG (U/L)	44	21	NA	9.2	3.3	3.3	10.9
α1M (mg/L)	52.9	36	NA	82.5	15.3	9.53	77.4
IGU (mg/L)	NA	9.21	NA	6.44	6.35	11.1	36.8
Size_k_ (cm*cm*cm)/ cortical thickness(cm)							
RK	9.4*4.3*3.2/1.6	10.6*4.7*4.7/1.2	8.0*4.9*4.5/1.5	11.5*4.1*3.7/1.5	10.0*4.9*4.7/1.7	10.6*5.4*4.8/1.5	10.5*5.6*4.8/1.4
LK	10.9*4.7*3.6/1.7	12.2*5.2*4.6/1.5	8.4*5.3*4.4/1.8	11.7*4.5*4.4/1.6	11.2*4.8*4.2/1.7	9.7*4.8*4.5/1.7	11.6*6.2*5.1/1.6
Duration of illness/ follow up(months)	3/NA	89/77	3/NA	27/NA	24/NA	20/2	6/NA

OMN: oligomeganephronia; M: male; F: female; BMI: body mass index; PrU: proteinuria; Scr: serum creatinine; BP: blood pressure; HeU: Hematuria; NEG: negative; NA: not available; GFR: glomerular filtration rate; eGFR: estimated GFR; RK: right kidney; LK: left kidney; Ccr: creatinine clearance rate; MAU: urinary microalbumin; TFU: urinary transferrin; NAG: N-acetyl-β-glucosaminidase; α1M: α1 microglobulin; IGU: urinary immunoglobulin; Size_k_: kidney size; *the fluctuation range during the disease.

The progression of illness was tracked before and after diagnosis for each case. The course of disease before diagnosis ranged from 3 to 27 months. After diagnosis by renal puncture, two cases (Case 2 and Case 6) were followed up for 77 months and 2 months, respectively. Overall, the course of the disease was traced from 3 to 89 months (mean 24.6 months) with Scr fluctuation ranged from 8 to 74 µmol/L. Case 2 had a longest duration of disease for 89 months. She had a slight elevation of Scr from the initial 116.9 µmol/L before biopsy to 169 µmol/L three years later and a declination of eGFR from 60.0 mL/min/1.73m^2^ to 36.6 mL/min/1.73m^2^. Another three years later, renal function decreased further with a Scr value of 184 µmol/L and eGFR of 32.4 mL/min/1.73m^2^. Proteinuria was trace during the period of follow-up. No one had progressed to ESRD.

### Pathological presentation of OMN cases

**Fig. 2:**
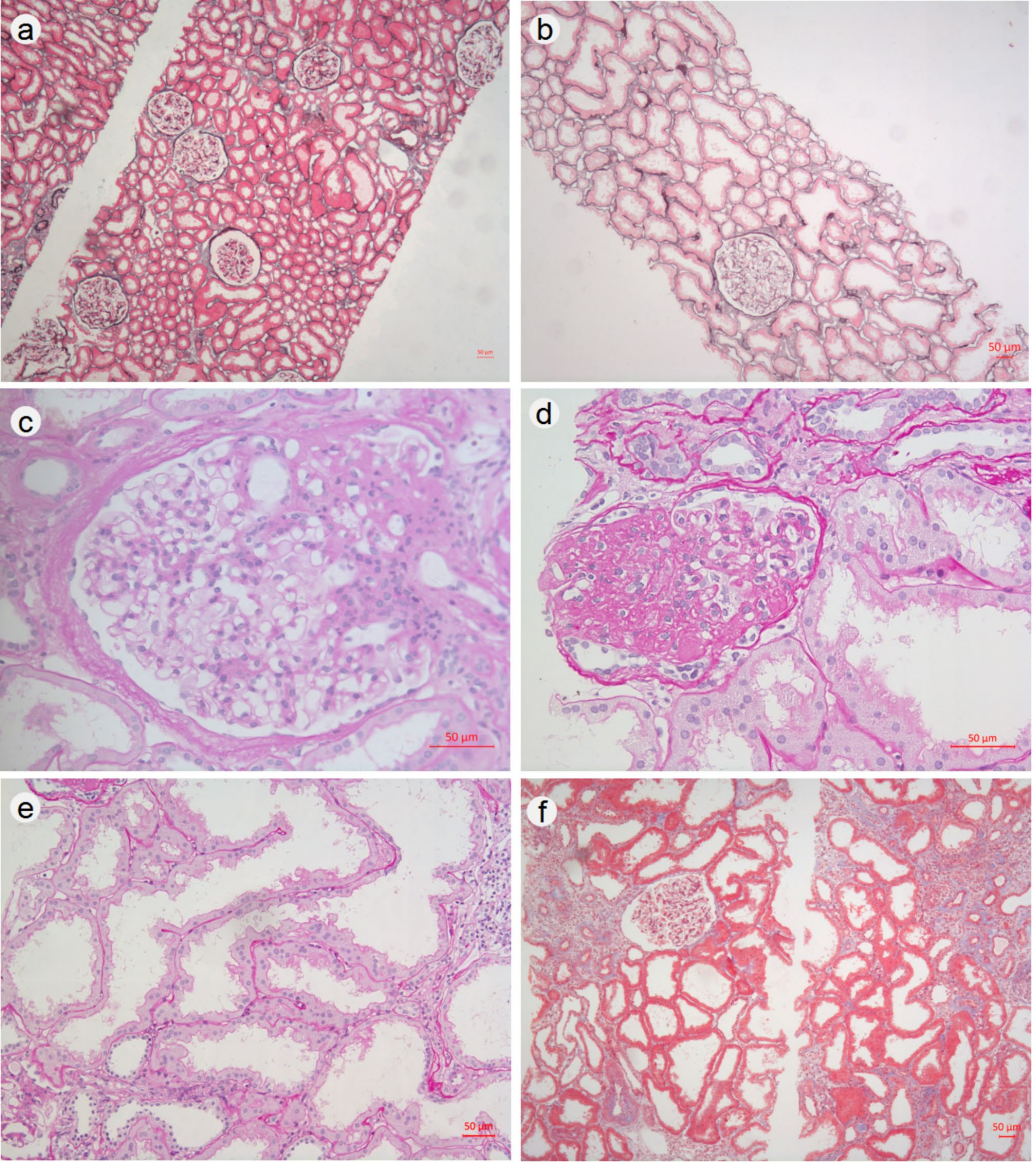
The pathological features of OMN cases by light microscope. By comparison with the control (a), OMN case showed much more sparsely distributed and enlarged glomerulus, and more dilated proximal tubules with some irregular in contour (b). Segmental sclerosis in OMN case was present with one at the vascular pole of a hypertrophic glomerulus with diameter of 270.89 μm (c), whereas the other could not be determined for the location where sclerosis started because of its invisible vascular pole and more extensive sclerosis (d). Markedly dilated and irregular proximal tubules lined by enlarged and hyperplastic epithelial cells in OMN were observed (e). Interstitial fibrosis was mild in OMN case (f). Silver methenamine, ×100 (a and b); Periodic acid-Schiff, ×400 (c and d); Hematoxylin and eosin, ×200 (e); Masson’s trichrome, ×100 (f). Scale bar, 50 μm

Pathological features of seven cases of OMN were exhibited in Table 2. Immunofluorescence microscopy for immunoglobulins and complements showed negative or weakly positive C3 and/or IgM in all cases. The area of renal cortex in the puncture tissue of the seven cases ranged from 9.13 to 31.19 mm^2^ which is sufficient for kidney biopsy. LM evaluation revealed much more sparsely distributed and enlarged patent glomeruli in OMN patients than that in the control (shown in Fig. 2.a,b). Segmental glomerular sclerosis was observed in six of seven cases (85.7%), and four of them (66.7%) were demonstrated to be of perihilar type (shown in Fig. 2.c, d). The density of glomeruli was 0.86 ± 0.29/mm^2^ (range 0.55–1.41/mm^2^). The mean glomerular diameter of seven cases was 229.73 μm (range 211.88–260.66 μm). The diameter of the largest glomerulus was 311.27 μm. The ratio of hypertrophic glomeruli in each case ranged from 40.0 to 90.9%. Statistical significance was not demonstrated in glomerular size or density between males and females (*P* > 0.05).

**Table 2:** Pathological features of the seven late-onset OMN cases

Case No.	Case 1	Case 2	Case 3	Case 4	Case 5	Case 6	Case 7
Immunoflorescence	IgM+	IgM+	C3+	IgM+, C3++	C3+	C3+	IgM+, C3++
Area of cortex (mm^2^)	9.13	13.52	14.89	31.19	16.78	10.78	15.11
Total number of glomeruli	7	8	21	17	15	9	15
Segmental sclerosis	1 (perihilar)	0	1 (perihilar)	1	2 (perihilar)	3	1 (perihilar)
Ischemic sclerosis	1	1	1	1	1	1	0
Density of glomeruli (/mm^2^)	0.77	0.59	1.41	0.55	0.89	0.83	0.99
Mean glomerular diameter (µm)	216.87	211.88	224.64	217.30	260.66	232.77	244.01
*Hypertrophic glomeruli (%)	40.0%	66.7%	60.0%	60.0%	90.9%	50.0%	66.7%
Mean short diameter of PT (µm)	74.55	86.89	77.69	100.53	81.27	109.08	77.11
*Dilated PT (%)	60.0%	80.6%	63.6%	89.4%	74.3%	94.2%	69.2%
Height of overlying epithelial cells of PT (µm)	11.82	12.27	11.94	15.41	15.93	12.78	16.16
Thickness of GBM (nm)	475.82	355.15	351.26	377.07	389.31	NA	411.59
Corrected FPW (nm)	657.84	558.89	639.21	556.39	634.24	NA	757.54

OMN: oligomeganephronia; PT: proximal tubule; GBM: glomerular basement membrane; FPW: foot process width; * By comparison with mean + 2SD in the control.

PT dilation was focal or diffuse and varied in intensity with irregular contour and some were lined by enlarged and focal multilayered epithelial cells (shown in Fig. 2.e). Both mean diameter of PT and height of epithelial cells lining PT in OMN cases was significantly greater than that of the age- and sex-matched control (*P* = 0.00001 and *P* = 0.0001), with the value of 87.01 ± 25.30 μm (range 36.44–235.63 μm) vs. 53.91 ± 2.25 μm (range 51.62–57.96 μm) and 13.76 ± 1.98 μm (range 11.82 ± 16.16 μm) vs. 9.23 ± 1.26 μm (range 7.73–11.66 μm) respectively. If the mean diameter of PT greater than upper cut-off value (the mean plus 2 times SD) in the control, namely 58.41 μm, was defined as dilated, the proportion of dilated tubules of OMN cases (range 60.0–94.2%) was much higher than that in the control (range 1.4–10.3%).

Mildly interstitial infiltration of lymphocytes and mononuclear cells were observed in all cases, with occasional eosinophils seen in one. In addition, interstitial fibrosis was minimal to mild degree (shown in Fig. 2.f). The interlobular arteries were almost normal with eccentric subendothelial hyalinosis in one case (Case 7).

**Fig. 3:**
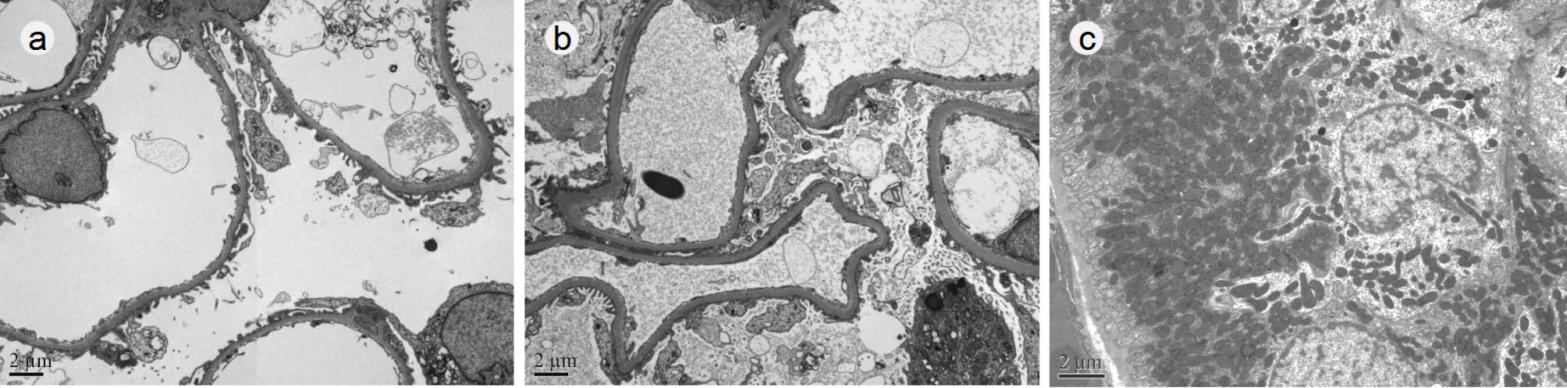
Ultrastructure features of OMN case by electron microscope. Foot process effacement of podocytes was mild (a) to moderate (b). In this hypertrophic tubular epithelial cell, small and crowded mitochondria were observed (c). Original magnification, ×6000 (a and b); ×8000 (c). Scale bar, 2 μm

Ultrastructural feature of glomeruli was obtained from six cases, except one case no glomerulus in the specimen was available for EM test. There was no abnormality in the thickness of GBM, with an average of 393.37 ± 42.12 nm (range 351.26–475.82 nm). No remarkable change was present in glomeruli, besides mild to moderate foot process effacement of podocytes (Fig. 3.a, b). The mean corrected FPW was 634.02 ± 74.19 nm (range 556.39–757.54 nm), which was wider than the normal range in the control (472.54 ± 37.27 nm) with a *P* value of 0.0002. In the tubular epithelial cells, numerous small mitochondria crowded in the cytoplasm were observed (Fig. 3.c).

## Discussion

OMN is generally referred to the classical cases, which is initially defined as a pediatric disease mainly occurs in infants or adolescents with a 3:1 male predominance and small kidneys. Patients are typically born premature or low birth weight for gestational age. It has been considered to be congenital and/or genetic. Most cases are sporadic and only a few are familiar, in twins or in siblings [12–14]. The clinical presentation starts with concentrating defects, such as dehydration, polyuria, and polydipsia, in the first few years of life [15]. Hyperfiltration with proteinuria develops later, followed by progressive renal failure. Hypertension is rarely present [16]. ESRD occurs months after birth or until early to mid-adolescence. Survival into adulthood without renal transplantation is unusual [17]. Obviously, the above features do not apply to the adult cases reported in the literature or in our series. Neither the concentrating defects nor distinctly decreased kidney size was observed in late-onset patients. In fact, the final diagnosis of OMN depends on pathological findings, which is even more essential for late-set ones. At present, there is still no clear quantitative index to diagnose late-onset OMN and no comprehensive summary of the features of this entity. It is generally accepted that the density and size of glomerulus are the main parameters to determine OMN.

Many factors were reported to be relevant to the number and size of glomeruli. Glomerular number decreased with age and was marginally lower in women [18, 19]. The size of human glomeruli has been reported to increase approximately seven-fold from infancy to adulthood [20, 21] and then decrease during senescence [22]. Male sex is more likely to have larger glomeruli than female. Interestingly, despite humans losing half of their nephrons from onset of adulthood to early seventies, compensatory hypertrophy of remaining glomeruli is not seen [23]. There is a direct correlation between glomerular number and birth weight with fewer glomeruli per unit area of cortex in low-birth weight neonates [20, 24]. The glomerular number and size in different racial groups is varied too [25]. Globally sclerotic glomeruli are smaller than nonsclerotic, and ischemic-appearing nonsclerotic glomeruli (capsule thickening, pericapsular fibrosis, and capillary wrinkling) may be smaller than nonischemic. Interstitial fibrosis > 25% is also associated with high frequency of global sclerosis with larger nonsclerotic glomeruli due to adaptive compensation [23]. Glomerular size is known to be larger with larger body surface area [22]. In addition, hypertensive diathesis of low nephron number and risk of chronic kidney disease (CKD) such as hypertension [26, 27], diabetes mellitus [28], smoking [29], family history of ESRD [19], higher serum uric acid level [19], and obesity [30, 31] are common factors associated with glomerular hypertrophy. Post-renal obstruction is another factor. In this study, we selected sex- and age-matched TBMN cases without the above factors leading to hypertrophic glomeruli as the control to investigate the normal range of glomerular density and size and then acquired the corresponding cut-off values for the diagnosis of OMN. To the best of our knowledge, this is the first report to set up the cut-off value for glomerular hypertrophy and rarity of OMN in adults.

Glomerular volume had previously been used to represent the size of glomeruli, which can be evaluated by several methods, such as the Cavalieri method, Weibel-Gomez method, principle dissector method, and the maximal planar area method [32]. Although the Cavalieri method is considered as the gold standard, it needs a relatively large tissue and is not applicable to kidney biopsy. The maximal planar area method, which is easy to perform, is the most commonly applied technique that can yield results closely related to those from the Cavalieri method. Measuring the diameter of capillary tuft is an improved maximal planar area method that is developed by Kambham et al. [33], and is more feasible and convenient in practice. By this means, we obtained the upper limit of glomerular size and lower limit of its density in the control, and they also served as the lower limit for glomerulomegaly (211.53 μm) and the upper limit of rarity of glomerular density (2.81/mm^2^). However, the cut-off value for the diagnostic criteria of OMN may be influenced by the calculating methods and ethnic differences.

Pathologically, the characteristics of late-onset OMN are as follows. Firstly, enlarged and sparsely distributed glomeruli are the main features. When the cut-off values of glomerular diameter and density applied, all nine cases suspicious of OMN met the diagnostic criteria for density while two not for size. However, all cases that met the criteria for hypertrophy also met that for rarity, and vice versa. Therefore, glomerular size may be a more stringent indicator. Based on current data, it is not clear whether late-onset OMN can be determined only by the size of glomeruli. However, this stringent criterion seems enough to screen in all our cases and easy to practice in daily life. Further test and verification of this idea with more cases is necessary. Secondly, segmental sclerosis (SS) was a common phenomenon (6/7) with most of them (4/6) surrounding the vascular pole. The ratio of SS was higher than that reported in the literature (3/7 for the former and 2/3 for the latter). As we know, larger glomeruli are evidence of hyperfiltration because they correlate with both higher single nephron GFR [34] and increased single nephron filtration capacity [35]. The resultant decrease in total glomerular filtration area leads to glomerular hypertension, hyperfiltration, and nephron enlargement that culminates in nephron sclerosis [16]. In addition, glomerulomegaly can itself lead to glomerulosclerosis due to podocyte injury caused by mechanical stretch [36]. On the other hand, perihilar sclerosis caused by high blood flow dynamics in afferent and efferent arterioles was additional evidence suggesting a state of hyperfiltration. Thirdly, dilated tubules were general and prominent, and the tubules were lined with hypertrophied epithelial cells. It has been reported in the literature [3] that hypertrophy was observed in the whole nephron of OMN cases, not only glomeruli but also tubules. However, there was no quantitative analysis performed in this respect. By measuring the short diameter of PT, we found markedly expanded lumen existed in all cases with a minimum percentage of 60%. By comparison with the control group, both degree of dilation of PT and height of overlying cells showed statistical significance (both *P* < 0.001). Hence, dilated PT may be another pathological feature as the diagnostic clue of OMN.

Clinically, by reviewing the sporadic reported cases in the literature and the seven cases in our series, the features of late-onset OMN were different from the classical early-onset type. First, late-onset OMN presented mainly in the third to fourth decade with a gentle male dominance, nine males and five females. Second, its clinical presentation is relatively temperate, with proteinuria and/or elevated creatinine of mild to moderate degree. Third, disease was progressing relatively slowly and no one diagnosed ESRD during the course of the disease. Fourth, the kidney size was either normal or only slightly reduced which is consistent with the fact that reduction in nephron mass in adult-onset OMN is much less than that in pediatric OMN [2]. In addition, it is notable that all patients in our series showed mild impaired renal tubular function. This is the first-time description in the literature of late-onset OMN.

As previously mentioned, many factors can lead to enlarged glomeruli and have similar pathological manifestations as late-onset OMN. For example, obesity-related glomerulopathy (ORG) is characterized by glomerulomegaly and focal segmental glomerulosclerosis (FSGS), particularly the perihilar variant, and may show significantly decreased glomerular density too [37, 38]. In our experience, ectatic renal tubules coated with hypertrophic epithelial cells can occasionally been observed in ORG patients. Consequently, late-onset OMN should be an exclusive diagnosis which could only be confirmed by quantitative pathological indices. Furthermore, the diagnosis may be more challenging due to the complexity of clinical and pathological manifestations when the lesion is in advanced stage. We do not propose a diagnosis of late-onset OMN for advanced lesions including those with IFTA more than 25%.

To date, the pathogenesis of OMN, especially the late-onset OMN, has not been elucidated clearly. Several abnormal phenomena had been found in early-onset cases but were controversial because no direct relationship was proved. These findings covered from inadequate embryonic development of the metanephric blastema [5], low birth weight and intrauterine growth retardation [39] to genetic abnormalities including *PAX2* mutation [2, 3], chromosome 4 deletion or mosaicism [40, 41], *RET1* gene mutation [42], and *HNF1B* gene mutation [43]. Heterozygous *PAX2* mutation had been found in one-third of children with OMN and therefore was considered to play a role in the etiology [2, 3]. For late-onset OMN, even less information was available. Genotype-phenotype correlations were investigated in only one case, and a novel heterozygous nonsense *PAX2* mutation in exon 4 was found [2]. Low birth weight or premature delivery was not existed in our cases.

## Conclusion

In conclusion, we investigated the diagnostic criteria of late-onset OMN by histomorphometric analysis for the first time and proposed that late-onset OMN is a special entity different from the early-onset OMN since their disparity in pathogenesis, clinical features and prognosis. Late-onset OMN is an exclusive diagnosis with unknown etiology characterized by typical pathological manifestations of enlarged and sparse glomeruli and expanded tubules with hypertrophic epithelial cells, and relatively slow clinical progress.

## Data Availability

The datasets used and/or analyzed during the current study are available from the corresponding author on reasonable request.

## List of Abbreviations

OMN: oligomeganephronia
IFTA: interstitial fibrosis and tubular atrophy
ESRD: end-stage renal disease
TBMN: thin basement membrane nephropathy
GBM: glomerular basement membrane
IF: immunofluorescence
LM: light microscopy
EM: electron microscopy
BMI: body mass index
Scr: serum creatinine
Ccr: creatinine clearance rate
NAG: N-acetyl-β-glucosaminidase
α1M: α1 microglobulin
GFR: glomerular filtration rate
eGFR: estimated GFR
PT: proximal tubules
TBM: tubular basement membrane
FPW: foot process width
SD: standard deviation
CKD: chronic kidney disease
SS: segmental sclerosis
ORG: obesity-related glomerulopathy
FSGS: focal segmental glomerulosclerosis
